# Radiological findings of COVID-19-related thromboembolic complications

**DOI:** 10.1186/s43055-021-00446-9

**Published:** 2021-04-08

**Authors:** Suzan Fouad Omar, Rehab Mohammed Habib, Abdelghany Mohammed Motawea

**Affiliations:** Menofia University, Faculty of Medicine, Shebin El-Kom, Egypt

## Abstract

**Background:**

The ongoing global pandemic of coronavirus disease 2019 (COVID-19) may cause, in addition to lung disease, a wide spectrum of non-respiratory complications. Among these are thromboembolic complications. The theories that explain the mechanism of thromboembolic complications of COVID-19 are accumulating rapidly, and in addition to the role of imaging for assessment of COVID-19 pneumonia, CT may be useful for identification of these complications, such as pulmonary embolism, ischaemic stroke, mesenteric ischaemia, and acro-ischaemia.

**Results:**

Thromboembolic manifestations were diagnosed in 10% of our patients (124 patients out of the total 1245 COVID-19 patients); 56 patients (45.2%) presented with pulmonary embolism, 32 patients (25.8%) presented with cerebrovascular manifestations, 17 patients (13.7%) presented with limb affection, and 19 patients (15.3%) presented with gastrointestinal thromboembolic complications.

Most of our patients had significant comorbidities; diabetes was found in 72 patients (58%), dyslipidemia in 72 patients (58%), smoking in 71 patients (57.3%), hypertension in 63 patients (50.8%), and morbid obesity in 40 patients (32.2%).

Thromboembolic events were diagnosed on admission in 41 patients (33.1%), during the first week in 61 patients (49.2%), and after the first week in 22 patients (17.7%).

**Conclusions:**

The incidence of thromboembolic complications in COVID-19 patients is relatively high resulting in a multisystem thrombotic disease. In addition to the crucial role of imaging for assessment of COVID-19 pneumonia, CT is important for assessment of the thromboembolic complications, such as pulmonary embolism, ischaemic stroke, mesenteric ischaemia, and peripheral ischaemia, especially in patients with elevated d-dimer levels and those with sudden clinical deterioration.

## Background

The ongoing global pandemic of coronavirus disease 2019 (COVID-19) may cause, in addition to lung disease, a wide range of non-respiratory complications due to involvement of organs by the virus or due to direct or indirect complications of this infection [1, 2]. Among these, thromboembolic complications due to abnormal coagulation were presented as an important issue in patients with COVID-19 infection and may occur in up to 31% of COVID-19 patients in intensive care unit (ICU) [3, 4].

Other organ systems are affected due to the marked affinity of the virus for the angiotensin-converting enzyme 2 (ACE2) receptors [5, 6]. Thus, tissues with high levels of ACE2 receptor are susceptible to direct infection [7]. These are most abundant in lung alveolar epithelial cells, enterocytes of the small intestine, and vascular endothelium [5].

Unlike hemorrhagic viruses (Ebola, Marburg...), Covid-19 is highly prothrombotic causing alterations in the coagulation cascade that leads to a progressive elevation of d-dimer correlated with the severity and extent of microthrombosis [8].

There is increasing evidence that thrombi are a major cause of multisystem organ dysfunction, including respiratory failure [9]. A number of studies showed that coagulation disorders related to COVID-19 are correlated with increased morbidity and mortality [10, 11].

COVID-19-associated thrombotic disease is suggested to be caused by various mechanisms including direct effects of COVID-19 through severe illness and hypoxia or severe inflammatory response or an indirect effect of infection related to investigational therapies used for treating COVID-19, which may have adverse drug interactions with antiplatelet agents and anticoagulants [12].

The theories that explain the mechanism of thromboembolic complications of COVID-19 are accumulating rapidly, and in addition to the role of CT chest for assessment of COVID-19 pneumonia, other imaging modalities may be useful for identification of these complications, such as pulmonary embolism, ischaemic stroke, mesenteric ischaemia, and acro-ischaemia.

## Aim of this study

The aim of this study was to estimate the prevalence of thromboembolic manifestations among COVID-19-positive patients and to describe the imaging findings of Covid-19-positive patients who presented with acute arterial or venous thromboembolic events in the pulmonary, cerebral, abdominal, or peripheral circulation.

## Methods

### Ethics approval and consent to participate

The study protocol was approved by the local Ethics Committee. All study procedures were performed in accordance with the ethical standards laid down in the 1964 Declaration of Helsinki and its later amendments. The ethics committee’s reference number is 9/2020RAD4.

All patients included in this study were informed that their clinical, laboratory, and radiological data will be used in the study. A written consent was taken from all patients.

### Study population

Between April and July 2020, we scanned 1245 COVID-19-positive patients, 124 of them (about 10%) had developed thromboembolic manifestations, pulmonary, abdominal, cerebral, or peripheral limbs.

The study included 73 males (58.9%) and 51 females (41.1%) with age ranged from 23 to 65 years old, (mean age 43.52 years ± 12.21 years).

*Inclusion criteria* included patients with confirmed Covid-19 infection who presented initially with thromboembolic symptoms or admitted to the hospital and then developed thromboembolic manifestations.

*Exclusion criteria* included patients with thromboembolic manifestations not confirmed to be COVID-19 positive and patients with chronic thromboembolic events.

### Imaging techniques

Patients underwent imaging for the suspected region according to the primary manifestations. Patients with suspected stroke underwent MRI brain with DWI according to the routine stroke protocol. Positive patients with acute stroke underwent MR cerebral angiography and venography.

The CT pulmonary and abdominal angiogram was performed after intravenous injection of 80–120 cc of iodinated contrast media at a flow rate of 3–5 ml/s followed by a 30 ml saline at the same rate. Region of interest was determined, and axial scans were taken during the contrast injection until a threshold enhancement and triggering a diagnostic scan. Indirect venography was done for the patients.

Patients with suspected peripheral arterial or venous acute arterial and/or venous thrombosis underwent duplex/Doppler examination of the affected limb followed by CT peripheral angiography for positive cases.

All CT scans were performed on a 128-slice multidetector CT scanner.

### Image analysis

The images were assessed for presence of vascular occlusion either arterial or venous.

Pulmonary CT angiography images were evaluated for location and extent of the thrombus, associated pulmonary manifestations of COVID-19, and presence of areas suggestive of lung infarction.

Abdominal CT angiography images were evaluated for evidence of arterial and/or venous thrombus (location and extent); involvement of IVC and portal, mesenteric, and iliac veins; and associated manifestations of bowel ischaemia (pneumatosis intestinale or portal vein gas) or intestinal obstruction. Evidence of solid organs infarction was evaluated.

Cerebral venogram and angiogram images were evaluated for location and extent of venous sinus thrombosis and evidence of acute arterial thrombosis and its location. Associated cerebral infarctions were evaluated for their extent and presence of hemorrhagic infarction. Evidence of diffusion restriction even with no definite vascular occlusion at angiography was considered thromboembolic events.

Duplex studies and peripheral angiography of the upper and lower limbs were evaluated for presence and extent of acute venous thrombosis and presence and extent of acute arterial occlusion.

### Data collection

Demographic and clinical data (presenting symptoms, risk factors, d-dimer values, and ICU admission) were collected from medical records. All data were compiled and analyzed.

## Results

**Fig. 1:**
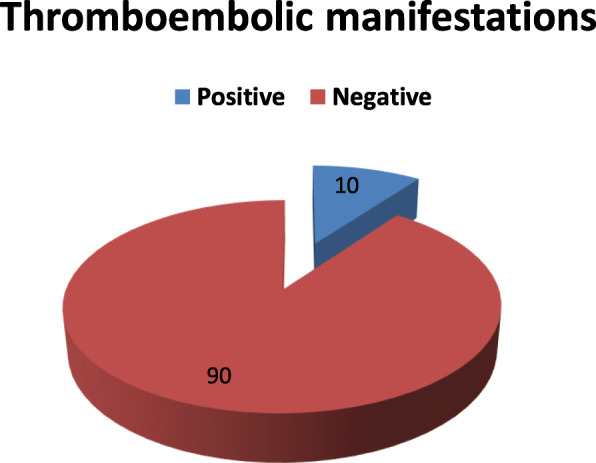
Prevalence of thromboembolic manifestations among COVID-19-positive patients

**Fig. 2:**
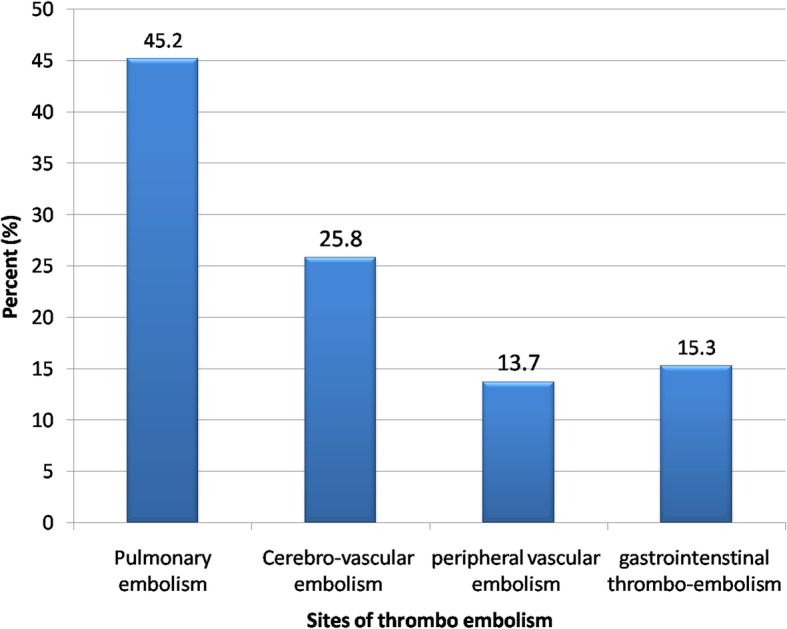
Sites of thromboembolism among COVID-19 patients

This study is observational cross-sectional study included 1245 COVID-19-positive patients, 124 of them (10%) developed thromboembolic manifestations (Fig. 1). Fifty-six patients (45.2%) had pulmonary embolism, 32 patients (25.8%) had cerebrovascular manifestations, 17 patients (13.7%) had peripheral vascular affection, and 19 patients (15.3%) had gastrointestinal thromboembolic complications (Fig. 2).

Patients with thromboembolic manifestations were 77 males (62.1%) and 47 females (37.9%) with age ranged from 23 to 65 years old (mean age 43.52 years ± 12.21 years). One or more comorbidities were found; diabetes was found in 72 patients (58%), dyslipidemia in 72 patients (58.1%), smoking in 71 patients (57.3%), hypertension in 63 patients (50.8%), and morbid obesity in 40 patients (32.3%).

The d-dimer level was very high notably in patients with pulmonary embolism (Table 1). Thromboembolic events were diagnosed on admission in 41 patients (33.1%), during the first week of admission in 61 patients (49.2%), and after the first week of admission in 22 patients (17.7%).

**Table 1:** Demographic data of the patients

	Pulmonary (***n*** = 56)		Cerebral (***n*** = 32)		Peripheral (***n*** = 17)		Abdominal (***n*** = 19)		Total (***n*** = 124)		***P*** value
	***N***	%	***n***	%	***n***	%	***n***	%	***n***	%	
**Mean age**	46.3 ± 10.53		45 ± 9.68		52.6 ± 7.9		41.7 ± 10.5		43.52 ± 12.21		0.01
**Sex**											
Male	**37**	66.1	**20**	62.5	**12**	70.6	**8**	42.1	77	62.1	0.25
Female	**19**	33.9	**12**	37.5	**5**	29.4	**11**	57.9	47	37.9	
**Risk factors**											
Hypertension	**28**	50	**23**	71.9	**6**	35.3	**6**	31.6	63	50.8	0.017
Diabetes	**35**	62.5	**25**	78.1	**9**	52.9	**3**	15.8	72	58.1	< 0.001
Dyslipidemia	**38**	67.8	**21**	65.6	**8**	47.1	**5**	26.3	72	58.1	0.009
Smoking	**35**	62.5	**18**	56.3	**10**	58.8	**8**	42.1	71	57.3	0.49
Morbid obesity	**15**	26.7	**11**	34.4	**6**	35.3	**8**	42.1	40	32.3	0.63
**Presence of pneumonia**	**54**	80.46	**25**	78.1	**10**	58.8	**5**	26.3	94	75.8	< 0.001
**Day of presentation**											
On admission	**34**	60.7	**3**	9.4	**2**	11.8	**2**	10.5	41	33.1	< 0.001
Within 1 week	**13**	23.2	**24**	75	**11**	64.7	**13**	68.4	61	49.2	
After 1 week	**9**	16.1	**5**	15.6	**4**	23.5	**4**	21.1	22	17.7	
**ICU admission**	**48**	85.7	**21**	65.6	**5**	29.4	**11**	57.9	85	68.5	< 0.001
**Hospital stay (average days)**	22 ± 3		18 ± 1		11 ± 2		14 ± 4		18.9 ± 3.7		< 0.001
**d-Dimer (average) ng/ml**	2765.45 ±169.11		1986.5 ± 202.1		2014.3 ± 108.04		2124.31 ± 155.49		2451.5 ± 174.5		< 0.001

Pulmonary embolism presented on admission in 34 patients (60.7%), 13 patients (23.2%) developed symptoms within the first week of admission, and 9 patients (16.1%) had pulmonary embolism after 1 week. The most presenting symptom was shortness of breath (52 patients 92.9%). Fourteen patients were presented with radiological findings that favor the evidence of lung infarction more than the classic features of COVID-19 pneumonia.

Three patients (9.4%) were presented with acute stroke. Twenty-four patients (75%) developed cerebrovascular symptoms within the first week of hospital admission for respiratory manifestations. Five patients (15.6) developed cerebrovascular symptoms after 1 week of admission (2 patients presented with sensory manifestations and found to have small thalamic infarction and 3 patients developed motor deficit). Twenty-five patients (78.1%) with cerebrovascular manifestations had associated pneumonia.

Ischaemic stroke has been associated with arterial thrombosis in 18 patients (40.6%) and venous sinus thrombosis in 9 patients (25%). Seven of them presented with hemorrhagic infarctions. Middle cerebral artery thrombosis was detected in 8 patients, internal carotid artery in 3 patients, anterior cerebral artery in 3 patients, basilar artery in 2 patients, and vertebral artery in 2 patients. Five patients (12.5%) presented with small infarcts identified only on MRI with no definite vascular involvement. Superior sagittal sinus thrombosis was identified in 5 patients, sigmoid sinus thrombosis in 2 patients, one patient presented with isolated straight sinus thrombosis, and one patient presented with internal jugular vein thrombosis.

Seventeen patients had peripheral thromboembolic manifestations; 9 patients (52.9%) had deep venous thrombosis of the lower limb, 2 patients (11.8%) had deep venous thrombosis of the upper limb, 5 patients (29.4%) had lower limb ischaemia, and one patient (5.9%) had upper limb ischaemia. All patients with deep venous thrombosis presented with edema of the affected limb (Table 2). Eleven patients (91.7%) of peripheral thromboembolic manifestations presented within the first week of admission.

**Table 2:** Presenting symptoms

Presenting symptoms	Pulmonary (***n*** = 56)		Cerebral (***n*** = 32)		Peripheral (***n*** = 17)		Abdominal (***n*** = 19)		Total (***n*** = 124)	
	***N***	%	***n***	%	***n***	%	***n***	%	***n***	%
**Fever**	**56**	100	**18**	56.25	**12**	70.6	**15**	78.9	101	81.5
**cough**	**49**	87.5	**12**	37.5	**6**	35.3	**4**	21	71	57.3
**Shortness of breath**	**52**	92.8	**–**	**–**	**11**	64.7	**4**	21	84	67.7
**Chest tightness**	**48**	85.7	**10**	31.25	**–**	**–**	**–**	**–**	58	46.8
**ECG changes**	**37**	66	**–**	**–**	**–**	**–**	**–**	**–**	37	29.8
**Abdominal pain**	**14**	25	**5**	15.6	**–**	**–**	**18**	94.7	37	29.8
**Diarrhea**	**–**	**–**	**2**		**–**	**–**	**8**	42.1	10	8.1
**Intestinal obstruction**	**–**	**–**	**–**	**–**	**–**	**–**	**2**	10.5	2	1.6
**Loss of consciousness**	**–**	**–**	**8**	25	**–**	**–**	**–**	**–**	8	6.5
**Motor weakness**	**–**	**–**	**16**	50	**–**	**–**	**–**		16	12.9
**Sensory loss**	**–**	**–**	**5**	15.6	**–**	**–**	**–**	**–**	5	4.0
**Headache**	**18**	32.1	**12**	37.5	**–**	**–**	**–**	**–**	30	24.2
**Limb edema**	**–**	**–**	**–**	**–**	**11**	64.7	**–**	**–**	11	8.9
**Acute claudicating pain**	**–**	**–**	**–**	**–**	**6**	35.3	**–**	**–**	6	4.8

Nineteen patients had abdominal thromboembolic manifestation; 17 venous thrombosis (89.5%) and 2 arterial embolism (10.5%). The most presenting symptom was abdominal pain (18 patients 94.7%). Isolated superior mesenteric vein thrombosis was detected in 4 patients (21%), isolated portal vein thrombosis occurred in 3 patients (15.8%), isolated inferior vein thrombosis was detected in 1 patients (5.3%), and combination of more than one vein thrombosis was detected in 9 patients (47.4%). Arterial thrombosis was detected in the superior mesenteric artery in 2 patients (10.5%), and both presented with bowel ischaemia and intestinal obstruction (Table 2).

## Discussion

The coagulopathy that goes with COVID-19 has gained increasing interest recently. The International Society of Thrombosis and Haemostasis reported that patients with severe coronavirus disease 2019 (COVID-19) have fulminant activation of coagulation and consumption of coagulation factors [13].

*In our study*, we found 124 patients (about 10%) with thromboembolic manifestations out of 1245 Covid-19-positive patients, 68.5% of them were admitted to the ICU. The rate of thromboembolism reported in the literature is variable. Some studies have reported thromboembolic rates in the range of 20–30% while others have reported rates as high as 40–70% [14]. The number of patients with thromboembolic manifestations in our study was less than that of Klok et al. [3] who observed thrombotic complications in 31% of ICU patients in a multicenter cohort of 180 patients admitted to the ICU of three Dutch hospitals, and that difference was due to the specific group of patients that they studied as all patients were in the ICU.

The presence of hypercoagulation and thromboembolic complications had been noted to correlate with disease severity and ICU admission [14]. *In our study*, 85 patients (68.5%) were admitted to the ICU.

**Fig. 3:**
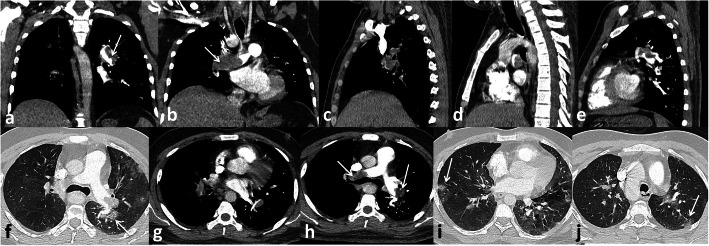
Male patient, 32 years old. Pulmonary embolism at the main pulmonary arteries (white arrows **a**, **b**, and **h**), ground-glass opacities of COVID-19 infection (white arrows **f** and **i**)

**Fig. 4:**
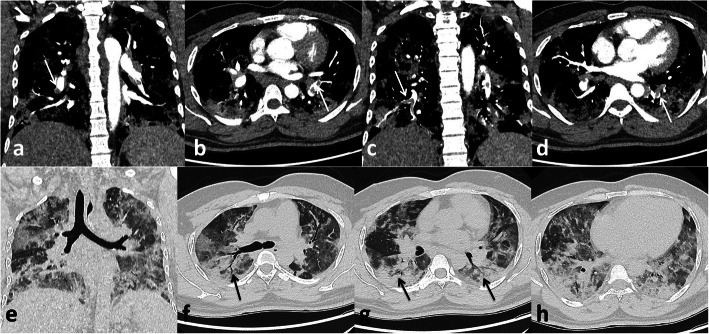
Male patient, 33 years old. Pulmonary embolism at segmental pulmonary arteries (white arrows **a, b, c, d**), bilateral consolidation patches of COVID-19 infection with air bronchogram (black arrows **f** and **g**)

**Fig. 5:**
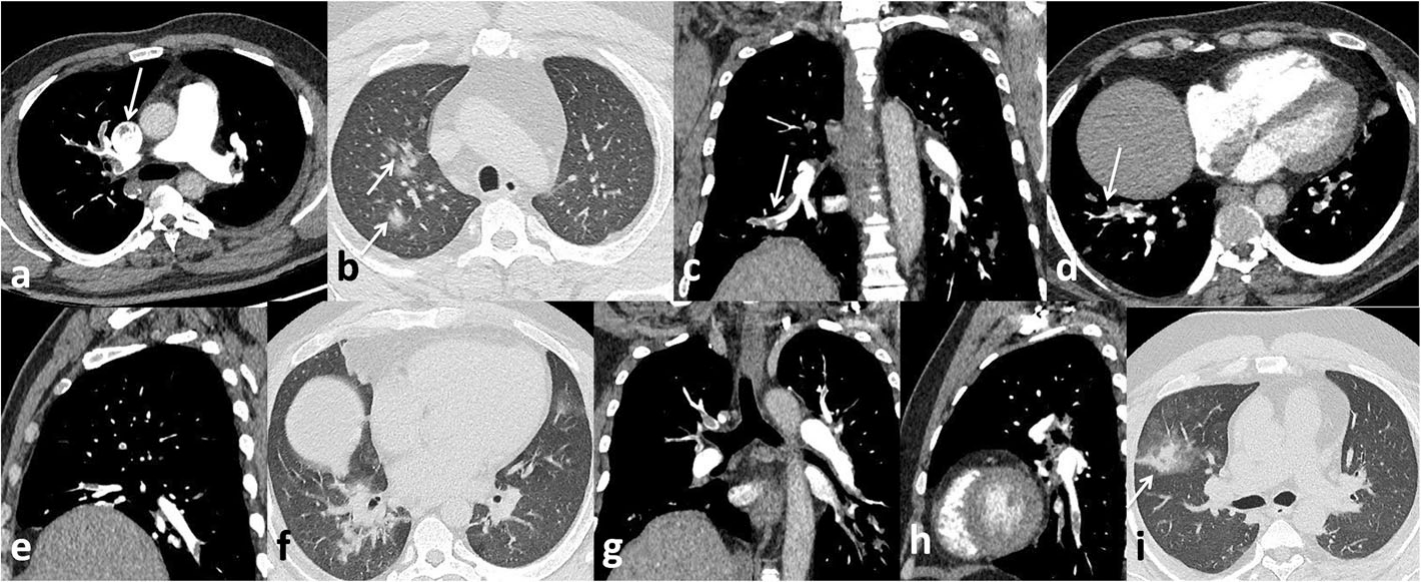
Male patient 52 years old, pulmonary embolism in right main pulmonary artery (white arrow in **a**), segmental artery (white arrow in **c**), andsubsegmental artery (white arrow in **d**), COVID-19 consolidation (white arrows in **b** and **i**)

In the current study, pulmonary embolism was the most common complication presented in 56 patients (45.2%) that was similar to Grillet et al. [15] who stated that pulmonary embolism is the most common thromboembolic complication of COVID-19; it has been presented radiologically in up to 30% of patients with COVID-19 on pulmonary CT angiography and in 14% of ICU patients diagnosed with COVID-19 pneumonia Figs. 3, 4 and 5.

In a meta-analysis conducted by Xiong et al. [16], they found that prothrombin time and d-dimer levels were significantly higher in patients with severe COVID-19 than in those with the mild disease. Lorant et al. [17] stated that COVID-19 patients with pulmonary embolism have higher d-dimer levels than those without pulmonary emboli and are more likely to be admitted to the ICU. They also reported that d-dimer > 2660 ng/mL has 100% sensitivity and 67% specificity for pulmonary embolism diagnosis prediction in COVID-19 patients, and that was the same in our study which found that highest d-dimer average value was found in patients with pulmonary embolism (average 2765.45 ± 169.11).

As a complication of pulmonary embolism, the lung infarction is rare due to the dual arterial supply of the lung, but it has been described in COVID-19 patients. On CT, lung infarction appears as ground-glass opacities in the early phases in unobstructed lung zones that represents pulmonary haemorrhage and peripheral wedge-shaped pulmonary consolidation [18, 19].

Therefore, the radiological findings of lung infarction from pulmonary embolism should be differentiated from GGO as well as consolidations of the COVID-19 pneumonia that may also show reverse a halo sign in about 4% of patients as this will positively impact the patient management. In non-enhanced CT, the presence of peripheral lung opacities with a reverse halo sign, dilatation of pulmonary trunk, and/or increased cardiac volume (particularly, enlargement of the right cardiac chamber) indicate risk of PE and may aid in the decision to perform CT pulmonary angiography to confirm or exclude pulmonary embolism [20]. Clinical parameters including highly elevated d-dimer levels, haemoptysis, and/or sudden worsening of respiratory function or chest pain should also be considered [21].

**Fig. 6:**
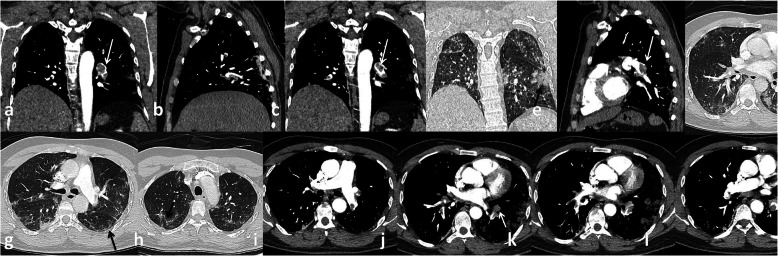
Male patient 43 years old with pulmonary emoblism at left main pulmonary artery (white arrows in **a**, **c**, **e**), subsegmental artery (white arrow in **j**), area of pulmonary infarction (black arrow in **d**), COVID-19 infection manifestations (black arrows in **f**, **g**, **h**)

**Fig. 7:**
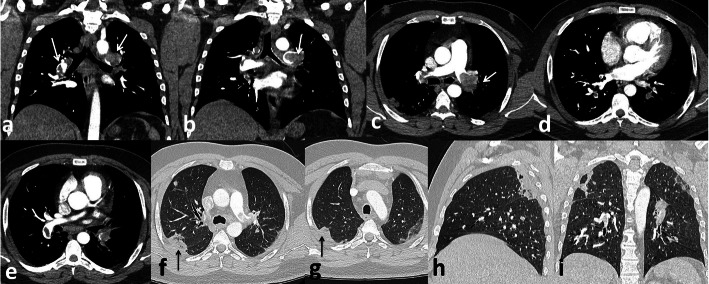
Male patient 63 years old. Pulmonary embolism at main pulmonary arteries (white arrows in **a**, **b**, **c**). Peripheral wedge shaped consolidation may represent pulmonary infarction black arrows in (**f** and **g**)

We have identified 14 patients with radiological findings that favor the evidence of lung infarction more than the classic GGO and consolidations of COVID-19. These patients had higher d-dimer levels with dilatation of the affected segment of the pulmonary artery. The peripheral wedge-shaped consolidation was associated with thrombus of the related subsegmental artery (Figs. 6 and  7).

**Fig. 8:**
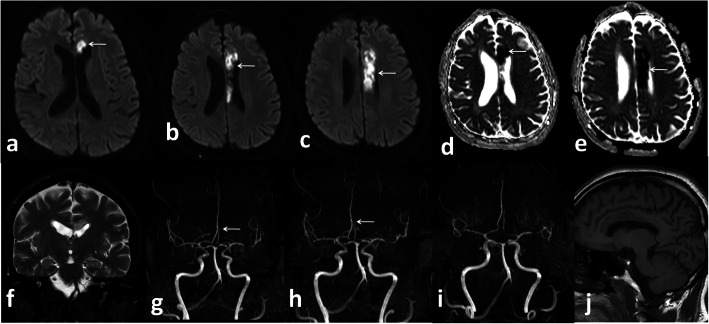
Female patient 54 years old. Left ACA territory infarction. DWI (white arrows in **a**,**b**,**c**), ADC map (white arrows in **d** and **e**), MRA defective A3 segment of left ACA (white arrows in **g** and **h**)

**Fig. 9:**
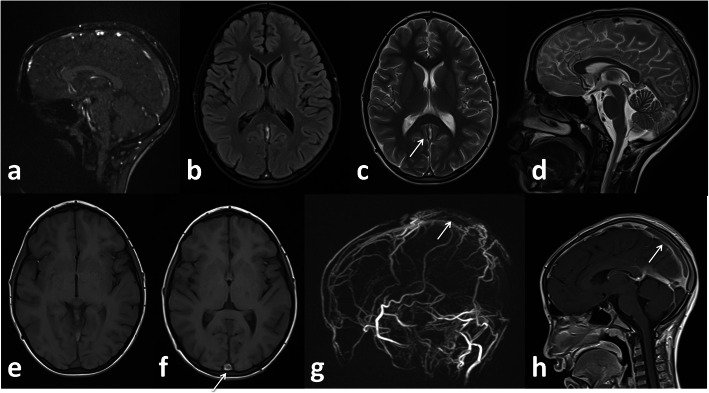
Female patient 42 years old. Superior sagittal sinus thrombosis (white arrows in **c**, **f**), filling defect in MRV (white arrow in **g**) and post contrast image (white arrow in **h**)

In the current study, we reported 32 patients with acute cerebrovascular manifestations, 27 (65.6%) of them had evidence of vascular occlusion (arterial in 18 patients Fig. 8 and venous sinus thrombosis in 9 patients Fig. 9) and 7 (21.88%) had haemorrhagic stroke. These results are close to those of Li et al. [22] who reported cerebrovascular manifestations for (6%) of 221 COVID-19 patients in a retrospective case series from Wuhan: 5% patients developed ischaemic strokes, < 1% intracerebral haemorrhage, and < 1% cerebral venous sinus thrombosis. However, the number of patients in our results was much less than the patients of Benussi et al. [23] who reported that (77%) of 55 COVID-19 patients admitted to one neurology unit with cerebrovascular disease, 35 of them had ischaemic stroke, three patients had haemorrhagic stroke, and five had transient ischaemic attacks. This difference is likely due to specific patient’s group that they studied as it was specialized neurology unit.

**Fig. 10:**
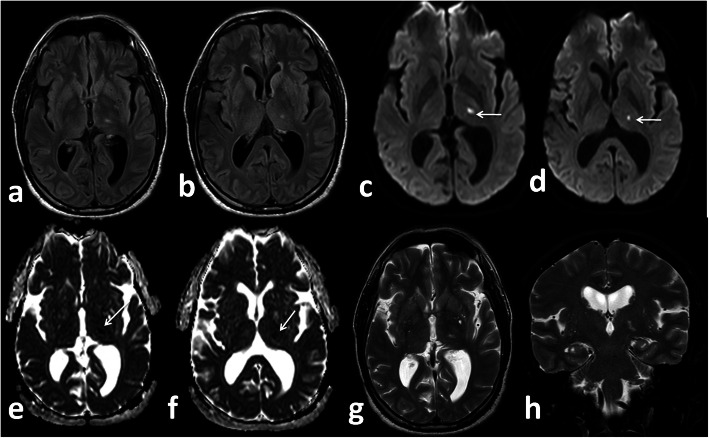
Male patient 48 years old presented with sensory loss. Left thalamic infarction. DWI (white arrow in **c** and **d**), ADC map (white arrows in **e** and **f**)

**Fig. 11:**
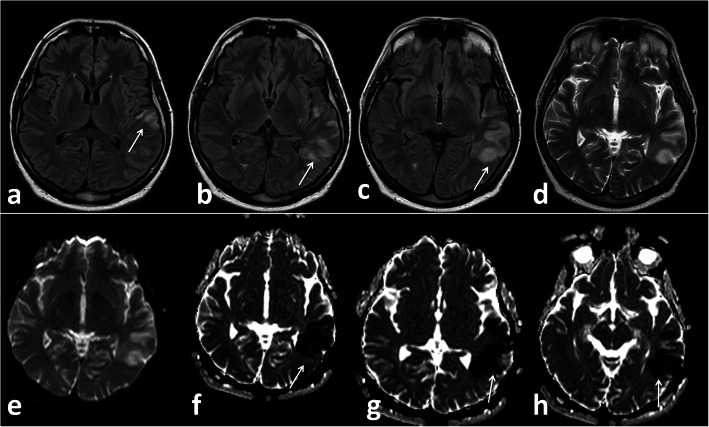
Female patient 62 years old presented with coma. Left temporal infaction (white arrows in **a**, **b**, **c**, **d**) restricted diffusion in ADC map (white arrows in **f**, **g**, **h**)

*In our study*, five patients (12.5%) presented with small infarcts identified only on MRI with no definite vascular involvement. Figures 10 and 11 Kandemirli et al. [24] reported that 44% of ICU COVID-19 patients with neurological symptoms showed abnormal findings on brain MRI studies.

**Fig. 12:**
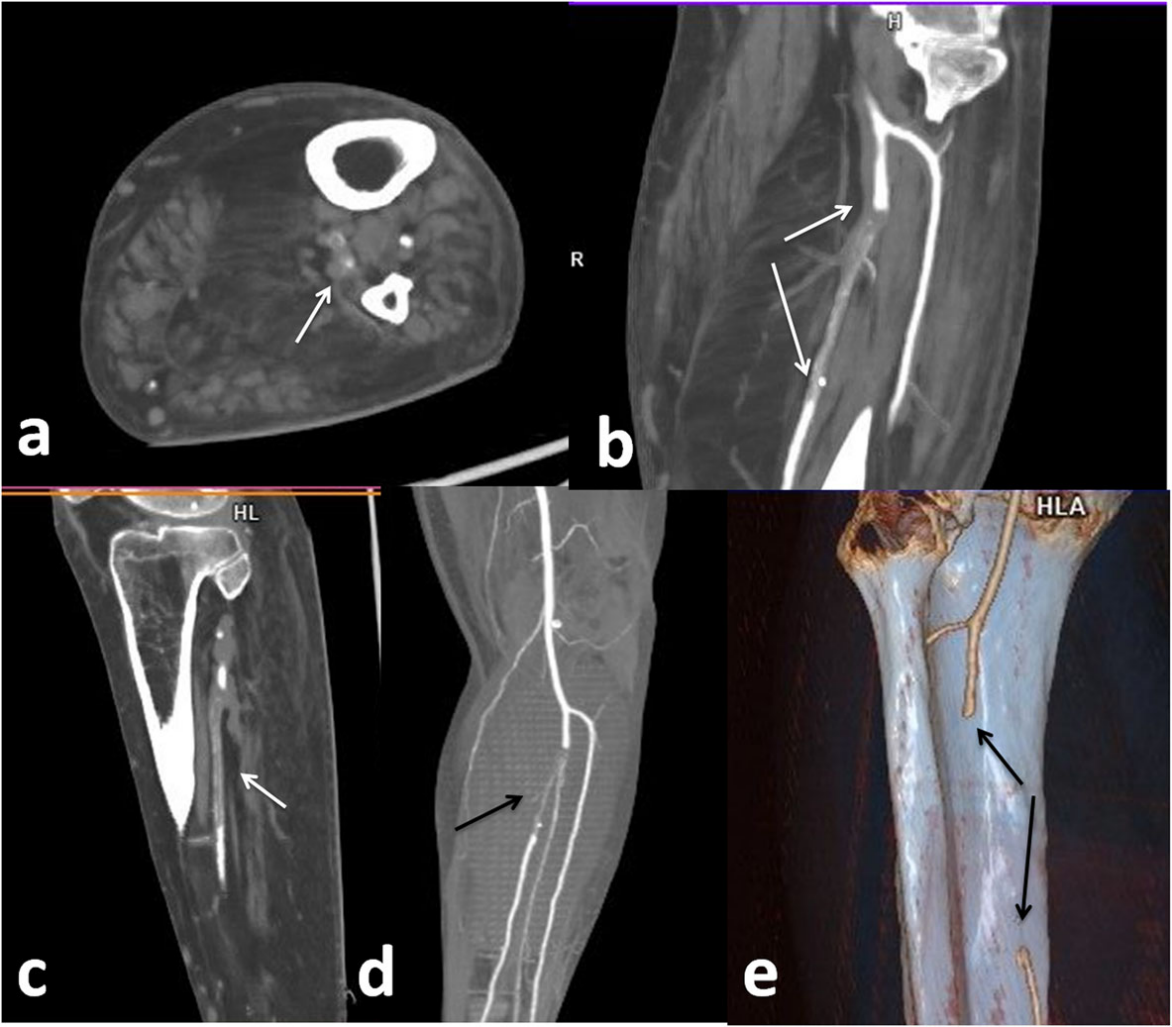
Female patient 52 years old. Acute thormbus of proximal seep peroneal artery (white arrows in **a**,**b**,**c**), CTA (black arrow in **d**), 3D reconstructed image (filling defect black arrows in **e**)

In a study done by Wichmannet et al. [25] on autopsy series, they found unsuspected deep vein thrombosis in the majority of COVID-19 patients, and that pulmonary embolism was the cause of death; also, Goldman et al. [26] stated that there is high incidence of arterial thrombosis in COVID-19 patients presenting with ischaemic leg symptoms (100% of cases in their cohort). They found that lower extremity arterial thrombosis associated with COVID-19 is characterized by greater thrombus burden and increased rate of amputation and death. *In our study*, 17 patients had peripheral thromboembolic manifestations, 9 patients (52.9%) had deep venous thrombosis of the lower limb, 2 patients (11.8%) had deep venous thrombosis of the upper limb, 5 patients (29.4%) had lower limb ischaemia Fig. 12, and one patient (5.9%) had upper limb ischaemia.

Bhayana et al. [27], in their study, described radiological abnormalities detected on abdominal CT in about 42% of COVID-19 patients as large or small bowel thickening, pneumatosis intestinale, portal vein gas, or bowel perforation, and also bowel ischaemia or necrosis was demonstrated in some of these patients who underwent surgery. In addition, acute infarction in abdominal solid organs was demonstrated in 4.8% of patients.

**Fig. 13:**
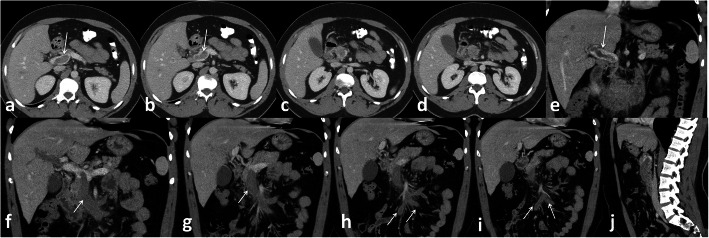
Male patient 35 years old, Thrombosis of portal vein (white arrows in **a**,**b**,**e**) extending into superior mesneteric vein ( arrows in **f** and **g**) and its territories (arrows in **h** and **i**)

*In the current study*, we reported 19 cases with gastrointestinal thromboembolic complications, 17 venous thrombosis (89.5%) and 2 arterial embolism (10.5%). The most presenting symptom was abdominal pain in 18 patients (94.7%). Isolated superior mesenteric vein thrombosis was detected in 4 patients (21%), isolated portal vein thrombosis occurred in 3 patients (15.8%), isolated inferior vein thrombosis was detected in 1 patients (5.3%), and combination of more than one vein thrombosis was detected in 9 patients (47.4%) Fig. 13. Arterial thrombosis was detected in the superior mesenteric artery in 2 patients (10.5%) and both of them presented with bowel ischaemia and intestinal obstruction.

## Conclusion

The incidence of thromboembolic complications in COVID-19 patients is relatively high, and the appropriate mechanism of these complications is the pro-coagulant pattern and the endothelial thrombo-inflammatory syndrome, which may result in multisystemic thrombotic disease.

In our study, the d-dimer level was more than 2600 ng/mL in 83 patients including all 56 patients with pulmonary emboli. So, in patients with high d-dimer levels on hospital admission or sudden clinical worsening, CT pulmonary angiography should be done to exclude pulmonary embolism

Also risk factors (diabetes, obesity, heavy smoking), clinical parameters, including haemoptysis, sudden worsening of respiratory function, acute abdomen and peripheral limb edema, or chest pain should raise the suspicions of thromboembolic complications.

So, in addition to the crucial golden role of CT chest for assessment of COVID-19 pneumonia, other imaging modalities (Duplex study, CT angiography, and MRI brain) are also important for the assessment of the thromboembolic complications, such as pulmonary emboli, cerebrovascular strokes, mesenteric vascular ischaemia, and peripheral ischaemia, especially in patients with high d-dimer levels and those with sudden clinical deterioration.

## Data Availability

All data and materials are available.

## Abbreviations

COVID-19: Coronavirus disease 2019
ACE2: Angiotensin-converting enzyme 2
CT: Computed tomography
ICU: Intensive care unit
MRI: Magnetic resonance imaging
DWI: Diffusion weighted imaging
